# In vivo biocompatibility of diamond-like carbon films containing TiO_2_ nanoparticles for biomedical applications

**DOI:** 10.1007/s10856-021-06596-6

**Published:** 2021-08-30

**Authors:** C. C. Wachesk, S. H. Seabra, T. A. T. Dos Santos, V. J. Trava-Airoldi, A. O. Lobo, F. R. Marciano

**Affiliations:** 1 Laboratory of Nanotechnology and Toxicology, Department of Science and Technology, UNIFESP—Federal University of São Paulo, São José dos Campos, São Paulo, SP Brazil; 2 Associated Laboratory of Sensors and Materials, INPE—National Institute for Space Research, São José dos Campos, São Paulo, SP Brazil; 3 Technology Laboratory of Biochemistry and Microscopy, UEZO—Universidade Estadual da Zona Oeste, Rio de Janeiro, RJ Brazil; 4 Laboratory of Cell Biology and Tissue, UENF—State University of Northern Rio de Janeiro, Campos dos Goytacazes, Rio de Janeiro, RJ Brazil; 5 Centro Universitário IBMR, Rio de Janeiro, RJ Brazil; 6 LIMAV-Interdisciplinary Laboratory for Advanced Materials, Materials Science & Engineering Graduate Program, UFPI—Federal University of Piaui, 64049-550 Teresina, PI Brazil; 7 Department of Physics, UFPI—Federal University of Piaui, 64049-550 Teresina, PI Brazil

## Abstract

Hybrid diamond-like carbon (DLC) with incorporated titanium dioxide (TiO_2_) nanoparticle coatings have low friction coefficient, high wear resistance, high hardness, biocompatibility, and high chemical stability. They could be employed to modify biomedical alloys surfaces for numerous applications in biomedical engineering. Here we investigate for the first time the in vivo inflammatory process of DLC coatings with incorporated TiO_2_ nanoparticles. TiO_2_-DLC films were grown on AISI 316 stainless-steel substrates using plasma-enhanced chemical vapor deposition. The coated substrates were implanted in CF1 mice peritoneum. The in vivo cytotoxicity and biocompatibility of the samples were analyzed from macrophage lavage. Analysis in the first weeks after implantation could be helpful to evaluate the acute cytotoxicity generated after a possible inflammatory process. The in vivo results showed no inflammatory process. A significant increase in nitric oxide production on the uncoated substrates was confirmed through cytometry, and the coated substrates demonstrated biocompatibility. The presence of TiO_2_ nanoparticles enhanced the wound healing activity, due to their astringent and antimicrobial properties. DLC and TiO_2_-DLC coatings were considered biocompatible, and the presence of TiO_2_ nanoparticles reduced the inflammatory reactions, increasing DLC biocompatibility.

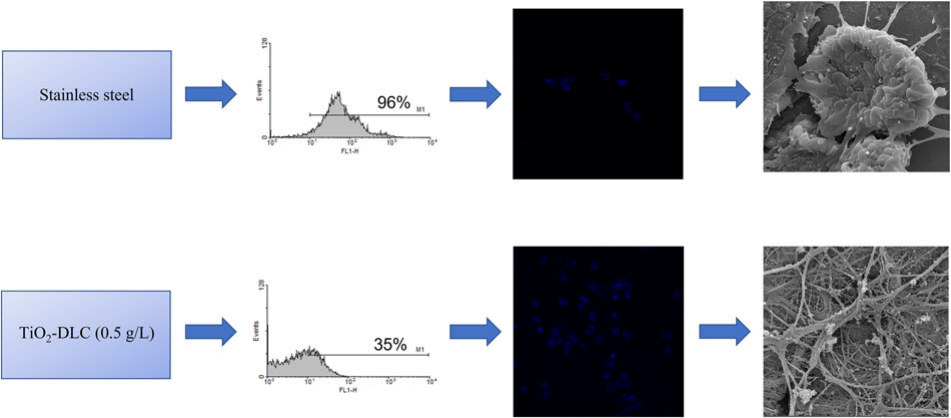

## Introduction

Medical implants are designed to ensure safety, for replacing or improving a lost or reduced function, for example, metal-on-metal hip implants or electronic devices for life-critical systems [1–4]. However, metal debris can trigger the host’s inflammatory response by activating the immune cells in the peri-implant space [4]. Furthermore, after 8.5 years of implantation, about half of the hip joints had to be revised due to aseptic loosening [5, 6]. In addition, a hospital charged an average of $34,328 for routine maintenance of acetabular and/or femoral components in total hip arthroplasties [7].

Over the last two decades, the potential use of diamond-like carbon (DLC) films for implant coatings has been extensively studied [5, 6, 8]. DLC coatings can impart low friction coefficient, high wear resistance, high hardness, good biocompatibility, and high chemical stability to the metallic surfaces of the implants, preventing osteolysis induced by worn particles [5, 6]. The studies have demonstrated the potential of DLC to control the inflammatory process, improve cell adhesion after incorporation of different nanoparticles, enhance bioactivity properties, and reduce bacteria adhesion and growth [5, 6, 9, 10]. However, few papers have conducted in vivo studies with DLC films [11–15].

The absence of computer models to predict the synergism between materials and the body makes it difficult to predict the long-term use of implants [1]. Therefore, in vivo tests need to be performed to avoid adverse tissue reactions such as infection and inflammation [16]. DLC films have biocompatibility and can facilitate osteogenesis after implantation [17–19]. Allen et al. [20] deposited DLC on polystyrene and implanted it intramuscularly in rats. Cytotoxicity within 90 days was not observed in vitro using human osteoblast cells or in vivo [20]. Evaluation of biomedical alloys using an in vivo model can provide an understanding of the inflammatory process associated with tissue formation and growth. Severe local inflammatory reaction after implantation is normally observed for several days. However, the implanted material should not be destroyed or phagocytosed, which would start a chronic phase of inflammation, characterized by the recruitment and permanence of exacerbated amounts of lymphocytes and macrophages [21, 22]. The next stage is fibroblast recruitment to produce collagen and then form a fibrous capsule between tissue and implant surface. If the material surface does not react with the tissue, a chronic inflammatory process could be initiated (due to the presence of granuloma) [23, 24].

The incorporation of titanium dioxide (TiO_2_) nanoparticles in DLC films improved fibroblast adhesion and decreased biofilm of gram-positive and negative bacteria due to photocatalytic activities, exhibiting the self-cleaning effect [8, 25–27]. The in vivo implantation could be helpful to promote tissue growth without granuloma presence. However, in vivo tests had not been performed yet. Hence, for the first time, we investigated the influence of DLC films containing incorporated TiO_2_ nanoparticles using an in vivo animal model. Analysis of the first weeks after implantation could be helpful to evaluate the acute cytotoxicity generate after a possible inflammatory process; therefore, possible in vivo inflammatory process and tissue integration of TiO_2_-DLC films growth on stainless-steel alloy were researched for 4 weeks.

## Experimental procedures

Polished AISI 316 L stainless-steel substrates (5 mm diameter, 1 mm thickness) were coated with DLC and TiO_2_-DLC films using plasma-enhanced chemical vapor deposition. The TiO_2_-DLC films were produced in two different concentrations (0.1 and 0.5 g/L) from TiO_2_ nanoparticles, Evonik Degussa P25 (21 nm diameter, in anatase crystalline phase). Details about the experimental methodology can be found in our previous manuscript [25, 26].

### In vivo experiments

The uncoated and coated (DLC and TiO_2_-DLC) samples were inserted surgically into the peritoneal cavity of 60 CF1 mice. In vivo experiments were conducted at 7, 15, and 30 days after the insertion. All in vivo experiments were approved by the experimental ethics committee in strict accordance with Brazilian Law (#11794, from October 8, 2014). The study was submitted to and approved by the Animal Use Ethics Committee of the State University of North Fluminense (http://www.clinicauenf.org/ceua4/), registered under the number 98.

The animals were divided into 5 groups, which were subdivided into 3 distinct time periods of 7, 15, and 30 days. Group 1, stainless-steel; Group 2, DLC; Group 3, TiO_2_-DLC (0.1 g/L); Group 4, TiO_2_-DLC (0.5 g/L); and Group 5, served as the control group and received glass slides. Only 3 mice were subjected to this implant, which was removed after 30 days. There was no control experiment (Group 5) for 7 and 15 days, to maintain the integrity of the animals.

For the surgical procedure, the animals were first weighed and anesthetized intramuscularly with a 1:1 solution of Ketamine (10% solution) and Xylazine (2% solution) at a dose of 0.10 mL/100 g of body weight. The site was cleaned with 70% ethyl alcohol. The samples were implanted in the peritoneum region of the CF1 mice on a small incision (~1 cm) made with a scalpel blade (number 15 and with 5 mm width). After the implantation, muscle tissues and skin were sutured with Vycril thread (Ethicon/J&J No. 4) following a standardized surgical procedure. The experiments were performed in triplicate (there were 3 animals in each group, one implant per animal, then three animals per group were sacrificed each time). After 7, 15, and 30 days, the mice were euthanized with CO_2_ and the implants were surgically removed.

### Peritoneal macrophages of mice

The obtained peritoneal lavage cells were labeled with F4/80 marker. This specific marker is used to identify phagocytes and macrophages. In mice, monocytes can be characterized by flow cytometry due to their low granularity and expression of molecules, such as antigen receptors recognized by the antibody F4/80 [28].

After the removal of implants, the peritoneal cavity was washed with 5 mL DMEM (Dulbelco’s modified Eagle’s medium/Sigma®—St. Louis, MO, USA) (at 4 °C) [29]. The fluid obtained was collected for analysis. Adhered cells were fixed and prepared for cytometry, nitrite dosage analysis, and scanning electron microscopy (SEM).

Peritoneal cells were incubated, centrifuged, and resuspended in a DMEM medium (2 × 10^5^ cells/mL) to obtain adherent cells. Then, 180 µL of cell suspension was filled in each well of a 24-well plate. After 1-h incubation, the wells were washed to remove non-adherent cells, and the adherent macrophages were fixed with paraformaldehyde, for 24 and 48 h.

### Flow cytometry

For the quantification of viable cells, the obtained adherent macrophages were incubated in phosphate-buffered saline, PBS (10^5^ cells/mL). After the incubation, the PBS was removed, and the remaining cells at the bottom of the tube were resuspended in 500 mL of PBS, which was again removed, and the cells were collected for analysis in the cytometer. The staining was performed with anti-F4/80 (1:100) for 15 min in 400 mL of PBS. The BD FACSCalibur flow cytometer (Becton-Dickinson, San Jose, CA, USA) was used to acquire data that were analyzed using WinMDI 2.8 software (Becton-Dickinson, San Jose, CA, USA).

### Nitrite measurement

The production of nitric oxide (NO) was analyzed by the Griess method [30]. The evaluation of NO production was performed indirectly by the determination of nitrite in the supernatant of the macrophage cultures after 24 and 48 h. For this analysis, 50 mL of supernatant was mixed with the same volume of Griess reagent (1:1) in 96-well plates. After 10 min, the absorbance was measured at 540 nm in a microplate reader. The nitrite concentration was estimated using a pre-calibrated standard curve using sodium nitrite diluted in DMEM [31].

### iNOS expression by immunofluorescence

For the immunofluorescence analysis, the macrophages were washed with PBS containing 0.5 μL of Triton X-100 for 15 min. After washing, 500 µL of ammonium chloride was added to the pre-washed cells and left for 30 min. Then, the cells were washed again with PBS and bovine serum albumin (BSA/PBS). Two markers were used: 1:100 rabbit anti-mouse (primary) and 1:200 goat anti-rabbit (secondary). Next, 25 µL of the primary antibody was added to the washed cells, left for 1 h, and then washed with PBS. Next, 25 µL of the secondary antibody was added, left for 1 h in the darkness, and again washed with PBS. After this procedure, the slides were mounted with DAPI (4′,6-diamidino-2-phenylindole).

### Scanning electron microscopy

The slides were fixed in Karnovsky fixative (4% paraformaldehyde, 2.5% glutaraldehyde, sodium cacodylate buffer 0.1 M, pH 7.4), dehydrated in acetone (30%, 40%, 50%, 70%, and 100%), and coated with gold plating (25 nm thick). After this processing, the images were captured by a Jeol JSM 6490LV SEM microscope.

### Statistical analysis

The One-way Anova (Graph Pad Prism 6^®^) was used to analyze the statistical difference. The populations from the stainless-steel, DLC, and TiO_2_-DLC films were obtained by the normal distribution. *P*-values less than 0.05 were considered to indicate statistical differences.

## Results and discussion

**Fig. 1:**
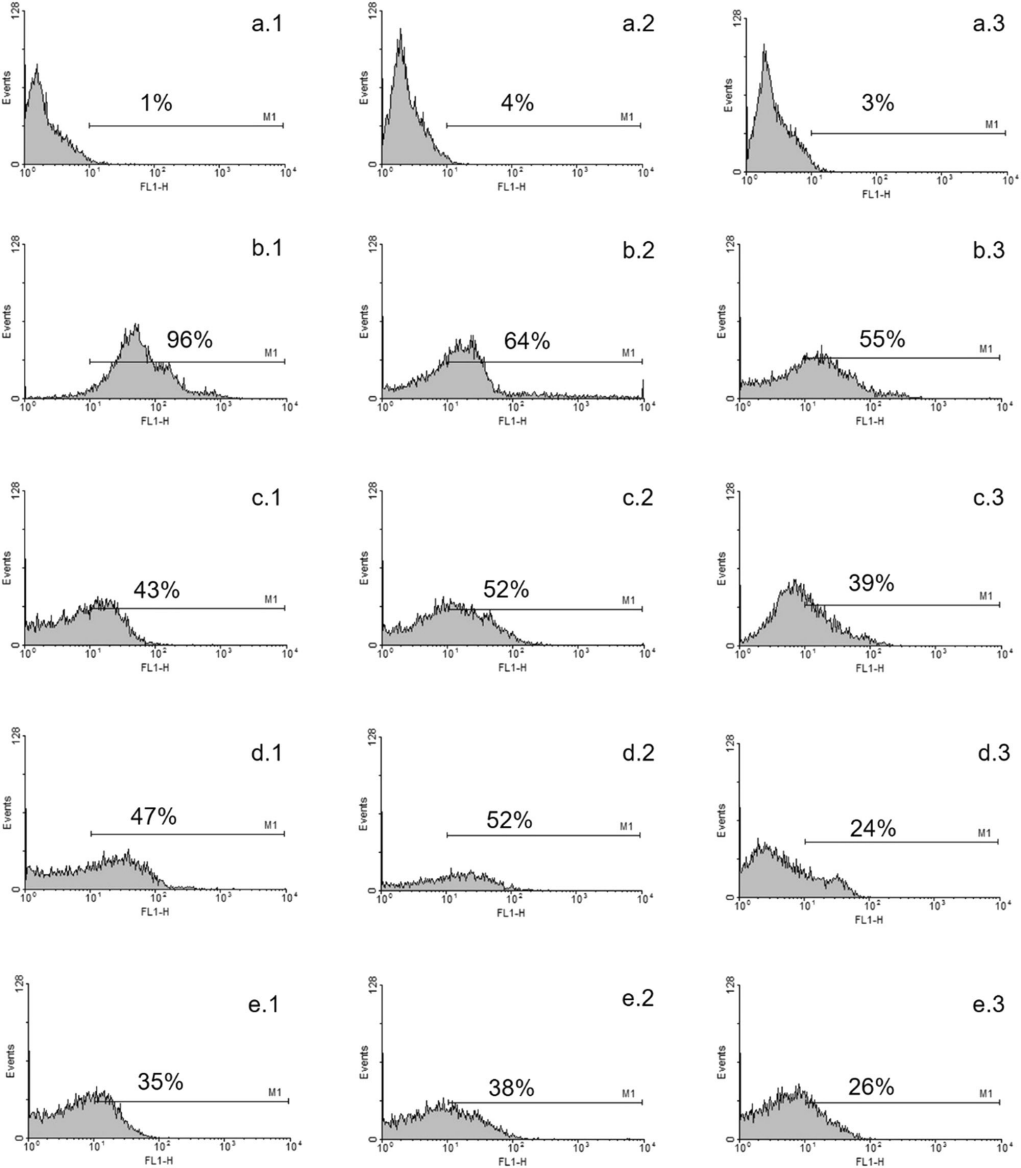
Histogram obtained by flow cytometry. **a** Control group with no implant (**a.1**) 7, (**a.2**) 15, and (**a.3**) 30 days after implantation. **b** Stainless-steel implants after (**b.1**) 7, (**b.2**) 15, and (**b.3**) 30 days. **c** DLC coated implants after (**c.1**) 7, (**c.2**) 15, and (**c.3**) 30 days. **d** TiO_2_-DLC (0.1 g/L) coated implants after (**d.1**) 7, (**d.2**) 15, and (**d.3**) 30 days. **e** TiO_2_-DLC (0.5 g/L) coated implants after (**e.1**) 7, (**e.2**) 15, and (**e.3**) 30 days

**Fig. 2:**
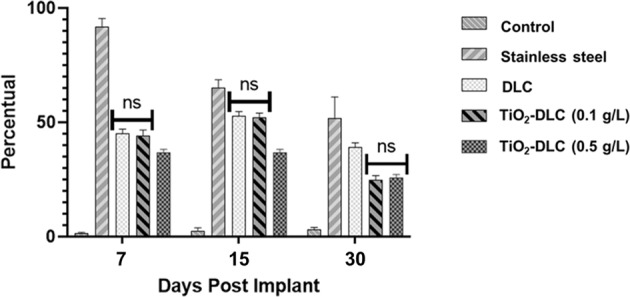
Percentual of F4/80—positive cells obtained by peritoneal lavage of different groups post-implant. Not was significative difference between the groups DLC and DLC/TiO_2_ (0.1 g/L). Data significance performed by ANOVA analysis

Macrophages are very important in the biological performance of biomaterials [32]. Figure 1 shows the histogram of macrophage activation obtained by flow cytometry at 7, 15, and 30 days. The control group (Fig. 1a), with no implant and consequently no inflammation process, exhibited less than 5% count in all periods. In contrast, uncoated stainless-steel implants (Fig. 1b) presented significant signs of activation (96% in 7 days), which is indicative of the inflammatory process. Macrophage activation decreased at 15 (Fig. 1b.2) and 30 days (Fig. 1b.3), reflecting the body’s response to healing itself. The presence of DLC films (Fig. 1c) decreased the number of counts. When TiO_2_ nanoparticles were present in the films, the number of counts tended to decrease, which was an indication of decreasing inflammatory process (Fig. 1e). Films with TiO_2_ nanoparticles achieved the best healing condition by 30 days (Fig. 1d.3, e.3). As nitrate measurement was performed indirectly, results of this method were not influenced by cell processing during in vitro culture, and results presented (Fig. 2) were from 3 repetitions ±SD.

The stainless-steel group suffered considerable inflammation, caused by the release of pro-inflammatory cytokines by macrophages and cytokine production by osteoblasts and fibroblasts [33]. The interaction of macrophages with DLC coatings did not induce any cellular inflammatory reaction. This result is in accordance with previous in vitro studies [26, 27]. DLC coatings improved biocompatibility and led to healing. The presence of titanium dioxide, due to its astringent and antimicrobial properties, enhanced the wound healing activity [34].

**Fig. 3:**
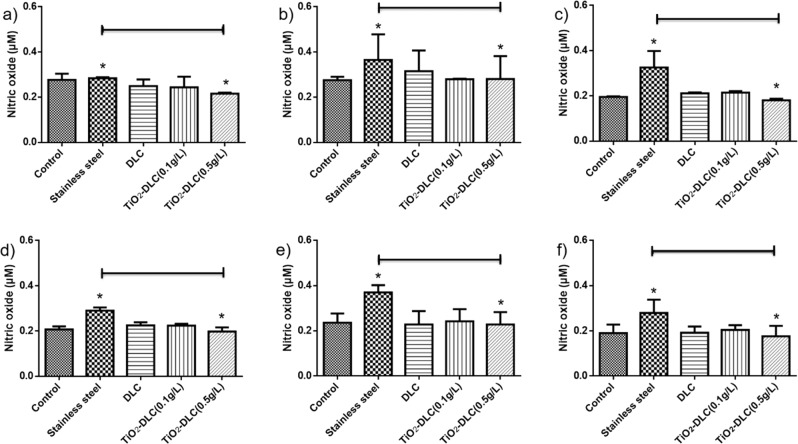
Nitric oxide in the supernatant of the macrophage cultures after 24 (**a**)–(**c**) and 48 h (**d**)–(**f**). Implants after (**a**) 7, (**b**) 15, and (**c**) 30 days, after 24 h of the macrophage cultures; and implants after (**d**) 7, (**e**) 15, and (**f**) 30 days, after 48 h of the macrophage cultures

When macrophages are activated by pathogenic microorganisms and foreign (cytotoxic) chemicals, they produce important immune mediators including nitric oxide [35]. Figure 3 shows the amount of nitric oxide after 24 (Fig. 3a–c) and 48 h (Fig. 3d–f), which exhibited no significant difference. A significative increase in NO production could be seen in the stainless-steel group at 15 (Fig. 3b, e) and 30 days (Fig. 3c, f), as compared to control (no implant) and those coated with DLC films. The excess NO suggests oxidative stress and mitochondrial dysfunction [31]. TiO_2_-DLC (0.5 g/L) implants decreased their NO concentration in the supernatant when compared to the uncoated implants. The presence of TiO_2_ nanoparticles in the films seems to decrease the cytotoxicity mediated by macrophages. These results corroborate the flow cytometry assays (Fig. 1).

**Fig. 4:**
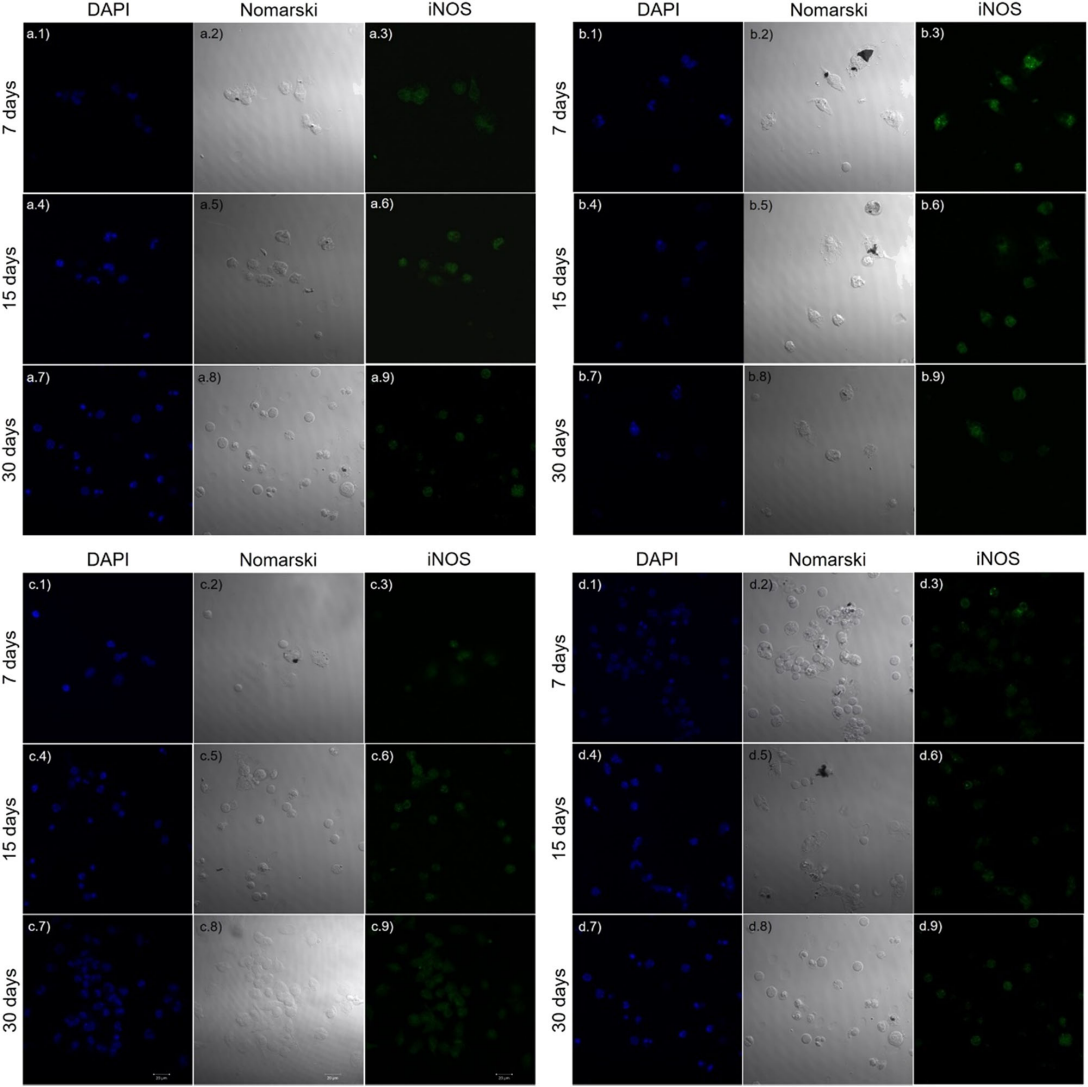
Peritoneal macrophages of CF1 mice that received (**a**) stainless-steel, (**b**) DLC, (**c**) TiO_2_-DLC (0.1 g/L); and (**d**) TiO_2_-DLC (0.5 g/L) implants. The cells were cultured for 48 h on glass coverslips at 7, 15, and 30 days. Cell nuclei were stained with DAPI (blue), in the left column; Differential interference contrast microscopy, also known as Nomarski microscopy, in the central column; and iNOS (green), in the right column. Magnification 63×

To support the presented results, immunofluorescence tests were performed on peritoneal macrophages obtained from the CF1 mice groups that received the implants at 7, 15, and 30 days (Fig. 4). The peritoneal cavity is one of the most employed sites to obtain macrophages for analysis and to model inflammation [36]. For this, the macrophages were cultured on glass coverslips for 24 and 48 h. The results at 48 h were more significant than those of 24 h. DAPI stained the nuclei of fixed cells and indicated cell integrity. To measure the amount of cytokines, endotoxin, and other inflammatory pre-stimuli, we performed iNOS [37].

Figure 4a illustrates the macrophage culture from the stainless-steel group. The cell nuclei (Fig. 4a.1, a.2) were slightly elongated, and the number of cells was lower than the others. The iNOS marking indicates the activation of macrophages (Fig. 4a.3). By 15 days (Fig. 4a.4, a.5), the cell nuclei remained slightly elongated, and the number of cells remained lower than the others. The considerable iNOS marking (Fig. 4a.6) indicated macrophage activation, which is evidence of an inflammatory process. By 30 days (Fig. 4a.7, a.8), the cell nuclei rounded, and the quantity of cells was higher. The iNOS marking (Fig. 4a.9) indicated macrophage activation, and thus an even greater inflammatory process.

In the macrophage culture from the DLC implant group by 7 days (Fig. 4b.1, b.2), the cell nuclei were slightly lengthened, and the number of cells was higher than the stainless-steel group. There were less iNOS marking (Fig. 4b.3) compared to the stainless-steel group. By 15 days (Fig. 4b.4, b.5), the cell nuclei became more rounded and elongated; the number of cells was similar to the stainless-steel group, but the marking for iNOS was less intense (Fig. 4b.6). By 30 days (Fig. 4b.7, b.8), the cell nuclei were round and elongated and the number of cells remained constant, but the marking for iNOS was much less intense (Fig. 4b.9).

Figure 4c illustrates cultures from the TiO_2_-DLC (0.1 g/L) implant group. In these cells (Fig. 4c.1, c.2), the nuclei remained round and the difference between cell number and cell morphology was significant compared to the others (Fig. 4a, b). The iNOS was not significant (Fig. 4c.3), which means the macrophages were not activated; therefore, no evidence of inflammation was found. At 15 and 30 days (Fig. 4c.4, c.5, c.7, c.8), the cell nuclei remained round and the number of cells was higher than in other groups. Moreover, the lack of marking for iNOS (Fig. 4c.6, c.9), indicated that macrophages were not activated, hence, no evidence of inflammation.

In the case of the cultures from the TiO_2_-DLC (0.5 g/L) group (Fig. 4d), at 7 and 15 days (Fig. 4d.1, d.2, d.4, d.5), the cell nuclei remained rounded and elongated with fewer cells than other groups at the same period. However, at 30 days (Fig. 4d.7, d.8), while the cell nuclei remained rounded, the quantity of cells was higher than the other groups. The marking for iNOS was not expressive in all the periods (Fig. 4d.3, d.6, d.9), indicating no activation of macrophages and no inflammation.

Nitrite measurement is a method widely used by different researchers to estimate the production of nitric oxide. In our experiment, peritoneal macrophages from animals in different experimental groups were plated, and the dosage was performed 24 and 48 h after plating, to obtain data about the activation of these macrophages. A control group was included so that we could compare the changes resulting from the response to the implants. Even if the experimental design could have general differences between the data obtained and the real ones, we compared the data with the control included in the experiment.

Expression of iNOS morphologically analyzes macrophages. If the macrophages are activated, they have the potential to be ideal targets for imaging inflammation [38]. In the cases without inflammation, surface structures (projections) and apparent phagocytes could continue as foreign material and did not cause inflammation.

Statistical analysis was performed referring to Figs. 3 and 4. However, it was not possible to identify significant differences related to the expression of iNOS and the indirect measurement of NO. These data were associated with the statistical data about the quantity of cells positive for F4/80; therefore, a greater quantity of these cells occurred in the samples collected from animals that received the stainless-steel implants. However, the NO production in these animals was not increased. This can be explained by the capacity of the immune system to modulate the production of NO by macrophages through signaling mechanisms, aiming at organism preservation. In addition, macrophages have different functions from initiation to the resolution of the process, in addition to participating in tissue regeneration [39, 40].

**Fig. 5:**
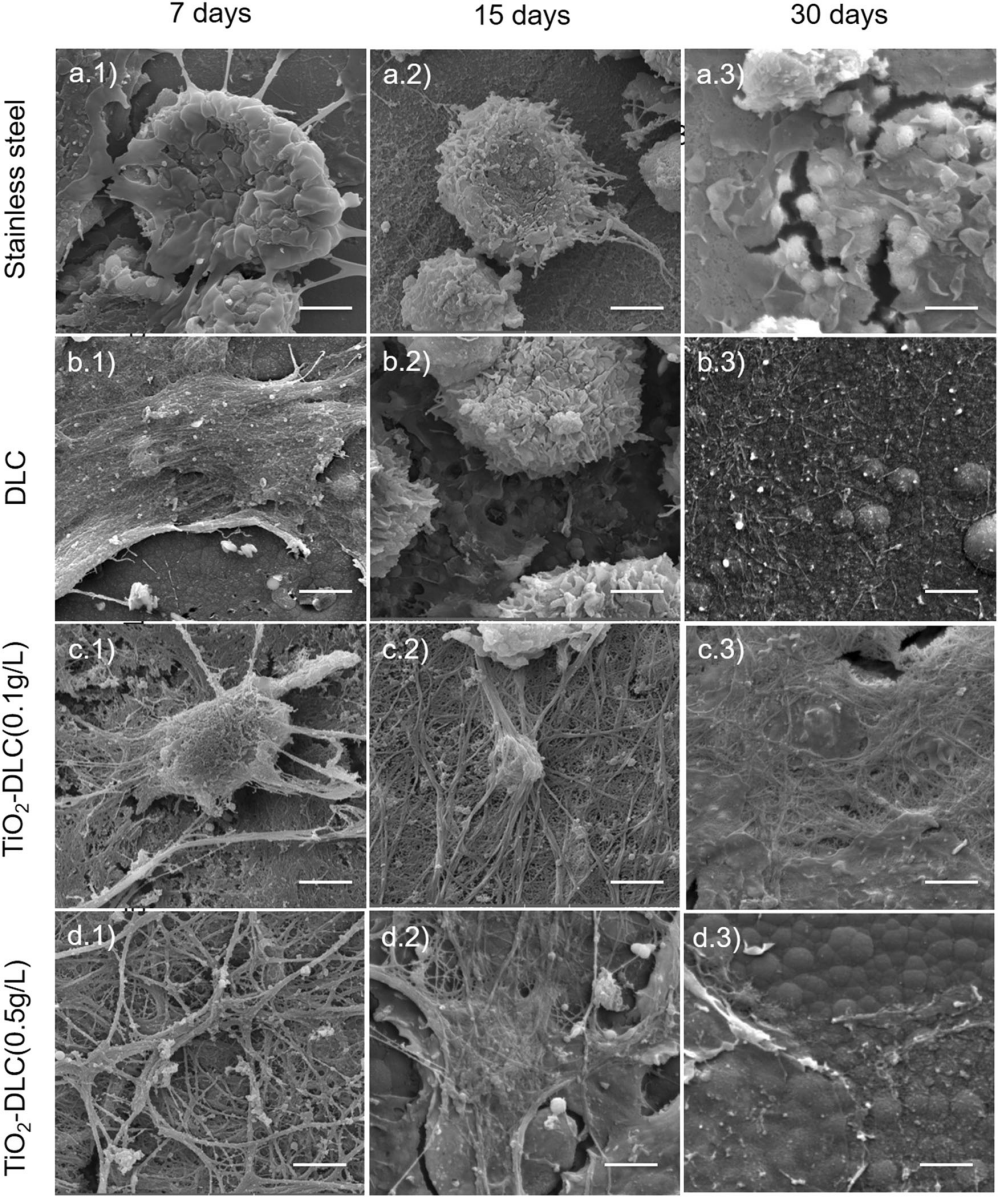
Scanning electron microscopy images of peritoneal macrophages from (**a**) stainless-steel, (**b**) DLC, (**c**) TiO_2_-DLC (0.1 g/L), and (**d**) TiO_2_-DLC (0.5 g/L) groups at (1) 7, (2) 15, and (3) 30 days. Each bar represents 2 µm. Magnification 20.0 k×

To confirm the results, cell morphology and adhesion were assessed by SEM. Figure 5 are SEM images from the macrophages at 7, 15, and 30 days. Macrophages are often elongated and quite flattened, with microvilli and/or cell extensions on their surface. They can establish points of adhesion and acquired fibroblastic characteristics.

In Fig. 5a, from stainless-steel implants, fewer adhered cells were observed at 7 days (Fig. 5a.1). At 15 days after implantation (Fig. 5a.2), more cells were visible than at 7 days, but only some of them have adhered. Likewise, at 30 days (Fig. 5a.3), even more, cells were visible, but still, very few have adhered to the substrate.

The image from the DLC group at 7 days (Fig. 5b.1) exhibits macrophages adhered to the substrate, confirming the adhesion and growth by the presence of filopodia and lamellipodia. By 15 days (Fig. 5b.2), the number of cells increased, and more adhered cells could be seen. By 30 days (Fig. 5b.3), the number of cells increased more, with strong adherence to the substrate, confirmed by the presence of filopodia and lamellipodia.

The presence of a pseudo confluent layer of elongated and adherent cells demonstrates very good adhesion and good spreading of the macrophages on the DLC and TiO_2_-DLC coatings (Fig. 5b, c, d). Cellular processes linking these cells were also observed in all of them.

Figure 5c–d are the images of cells from TiO_2_-DLC (0.1 g/L) and TiO_2_-DLC (0.5 g/L) implants. They had the same behavior. At 7 days (Fig. 5c.1, d.1), the presence of filopodia and lamellipodia were indicative of adhered cells. An increasing number of cells was observed at 15 days (Fig. 5c.2, d.2) when compared to 7 days. At 30 days after implantation (Fig. 5c.3, d.3), the number of cells remained constant, but more adherent.

These results indicated that the macrophages were not activated by the DLC and TiO_2_-DLC implants. Classically, activated macrophages appear to be the predominant cell type around 48 h after the initial injury [41]. The role of macrophages at this point is to phagocytose debris and apoptotic cells, which can facilitate wound healing or repair macrophages [42].

**Fig. 6:**
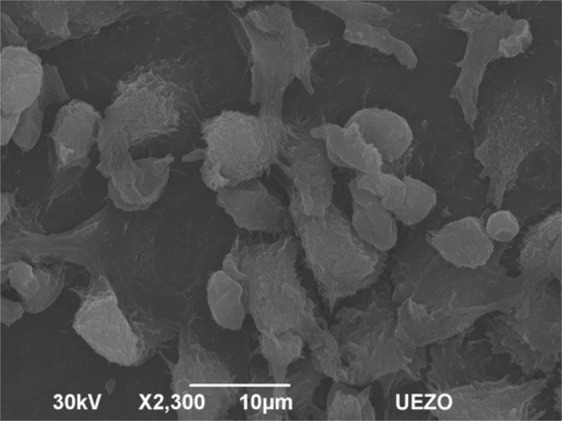
Micrography of peritoneal macrophages on glass coverslip implant at 30 days after implantation. Magnification 2.3 k×

When macrophages are activated, they influence other cells by releasing inflammatory mediators. The control was performed only in animals of the 30-day group (Fig. 6) because it is already known that glass coverslips cause a chronic inflammatory response (activate macrophages). With the glass coverslip implant, the number of cells was similar to that found in DLC and TiO_2_-DLC implants (Fig. 5). However, there was no cell adhesion to the substrate and no predominance of flat and scattered cells. In contrast, the DLC and TiO_2_-DLC implants showed good adhesion. The predominance of flat cells and spreading evidence increased cell adhesion due to TiO_2_ nanoparticles.

The biocompatibility of a material depends on its biological response to its insertion into the body [43]. This in vivo study demonstrated that the DLC and TiO_2_-DLC implants were biocompatible, as they were not cytotoxic. The incorporation of TiO_2_ in DLC films effectively increased cell viability compared to the other groups. The non-occurrence of any acute inflammatory response, as observed with flow cytometry and measurement of nitric oxide, evidences that the DLC and TiO_2_-DLC films were non-cytotoxic and the presence of TiO_2_ in DLC films increased the anti-inflammatory response.

## Conclusion

Here for the first time, the in vivo inflammatory process of DLC coatings containing TiO_2_ nanoparticles was investigated. The in vivo cytotoxicity and biocompatibility of the implants were analyzed from macrophage lavage. No infection was found in peritoneal macrophages of mice in contact with DLC and TiO_2_-DLC implants. The nitric oxide production significantly decreased in groups coated with DLC and TiO_2_-DLC films. The iNOS showed that the macrophages were not activated, proving that there was no inflammation. The presence of TiO_2_ nanoparticles enhanced the wound healing activity, due to its astringent and antimicrobial property, and increased cell adhesion.

Besides DLC and TiO_2_-DLC coatings were considered biocompatible, the presence of TiO_2_ nanoparticles reduced the inflammatory reactions increasing DLC biocompatibility. Our results are of great importance to demonstrate the applicability of TiO_2_-DLC as coatings of biomedical alloys.
